# Optimized Ultrasound-Assisted Extraction of Phenolic Compounds from *Polygonum cuspidatum*

**DOI:** 10.3390/molecules19010067

**Published:** 2013-12-20

**Authors:** Chia-Hung Kuo, Bao-Yuan Chen, Yung-Chuan Liu, Chieh-Ming J. Chang, Tzu-Shing Deng, Jiann-Hwa Chen, Chwen-Jen Shieh

**Affiliations:** 1 College of Tea and Food Science, Wuyi University, Fujian 354300, China; E-Mail: chkuo@wuyiu.edu.cn; 2 Graduate Institute of Molecular Biology, National Chung Hsing University, Taichung 402, Taiwan; E-Mail: boq609@gmail.com; 3 Department of Chemical Engineering, National Chung Hsing University, Taichung 402, Taiwan; E-Mails: ycliu@dragon.nchu.edu.tw (Y.-C.L.); cmchang@dragon.nchu.edu.tw (C.-M.J.C.); 4 Department of Agronomy, National Chung Hsing University, Taichung 402, Taiwan; E-Mail: tsdeng@dragon.nchu.edu.tw; 5 Biotechnology Center, National Chung Hsing University, Taichung 402, Taiwan

**Keywords:** ultrasound-assisted extraction, *Polygonum cuspidatum*, piceid, resveratrol, emodin, RSM

## Abstract

In this study the phenolic compounds piceid, resveratrol and emodin were extracted from *P. cuspidatum* roots using ultrasound-assisted extraction. Multiple response surface methodology was used to optimize the extraction conditions of these phenolic compounds. A three-factor and three-level Box-Behnken experimental design was employed to evaluate the effects of the operation parameters, including extraction temperature (30–70 °C), ethanol concentration (40%–80%), and ultrasonic power (90–150 W), on the extraction yields of piceid, resveratrol, and emodin. The statistical models built from multiple response surface methodology were developed for the estimation of the extraction yields of multi-phenolic components. Based on the model, the extraction yields of piceid, resveratrol, and emodin can be improved by controlling the extraction parameters. Under the optimum conditions, the extraction yields of piceid, resveratrol and emodin were 10.77 mg/g, 3.82 mg/g and 11.72 mg/g, respectively.

## 1. Introduction

*Polygonum cuspidatum*, also called Huzhang, belongs to the Polygonaceae family and is a perennial herb widely distributed in Asia and North America. The roots of *P. cuspidatum* are used as a traditional Chinese medicinal herb for the treatment of arthralgia, chronic bronchitis, jaundice, amenorrhea, hypertension, and hypercholesterolemia [1]. The known phenolic compounds found in the roots of *P. cuspidatum* mostly associated with these properties are resveratrol, piceid, and emodin [2,3]. Phenolic compounds can readily form resonance-stabilized phenoxy radicals that possess good antioxidant activities [4,5]. Resveratrol and piceid have been shown to possess various biological activities such as antioxidant, anti-inflammatory, anti-cancer, anti-aging and cardioprotective properties [6,7,8]. Emodin has been shown to possess anti-inflammatory, antibacterial, and antineoplastic activities [9,10,11,12].

Traditionally, the extraction of phenolic compounds was accomplished by Soxhlet extraction (heating in a solvent to reflux). However, the extraction operated at high temperature for a long time might degrade the extracted compounds, and it also has a high energy cost. Many studies have examined the extraction of piceid, resveratrol and emodin from *P. cuspidatum* by using various extraction techniques. Supercritical fluid with acetonitrile as modifier at 40 MPa, 100 °C has been used for the extraction of piceid, resveratrol and emodin and gave 0.22, 0.25, and 11.84 mg/g yield, respectively [13]. Microwave-assisted extraction of resveratrol using 1-butyl-3-methylimidazolium bromide solution has been reported to afford 2.65 mg/g yield of that compound [14]. Compared to these methods, ultrasound-assisted extraction has attracted much interest in the extraction of bioactive substances from plant materials. Ultrasound-assisted extraction is one of the most simple, inexpensive extraction systems and can be operated rapidly in a broad range of solvents for large-scale preparations suited for industrial purposes [15]. Pilot plant scale ultrasound-assisted extraction has been used for enrichment of phenolic compounds in olive oil [16] and extraction of polyphenols from apple pomace [17]. Besides, ultrasound-assisted extraction has been developed for the fast extraction of antioxidants from black soybean [18], lycopene from tomatoes [19], carotenoids from carrots [20], phenolic compounds from blackberry leaves [21], and flavonoids from several plants such as *Sparganii rhizoma* [22], *Prunella vulgaris* L. [23], and hawthorn seed [24]. Sonication is the production of sound waves that create cavitations in the liquid solution, subsequent collapse of the cavitations bubbles close to the plant material surface causes an increase in pressure and temperature, which will destroy the cell walls of the plant matrix and release the extractive compounds into the solution [25]. However, it should be kept in mind that some minor components are affected by ultrasound treatment and might be degraded after sonication [26,27].

Generally, ultrasound-assisted extraction is affected by several parameters, such as ultrasonic power, solvent concentration, and extraction temperature. In order to achieve higher extraction yields, a model for the optimization of the most relevant operational parameters is required. Response surface methodology (RSM) is a mathematical and statistical technique, that has been successfully applied to investigate the possible interactions and to optimize experimental variables in various processes [28,29]. Compared with a one-factor-at-a-time design, which is adopted most frequently in the literature, the experimental design and RSM were more efficient in reducing the number of experimental runs and time needed for investigating the optimal conditions for extraction. In this work, multiple RSM and Box-Behnken designs were employed to investigate the affinities between the extraction variables (solvent concentration, extraction temperature and ultrasonic power) and response (extraction yield %), and to obtain the optimal conditions for extraction of piceid, resveratrol, and emodin from *P. cuspidatum*.

## 2. Results and Discussion

### 2.1. Model Fitting

RSM is a useful statistical technique for investigating complex processes. Using this technique, optimal conditions for the extraction of multiple compounds can be obtained. To investigate the relationship between the extraction conditions (temperature, ethanol concentration, and ultrasonic power) and the extraction yields of the three compounds (piceid, resveratrol, and emodin), multiple RSM combined with a three-level, three-factor Box-Behnken design was employed in this study. 

**Table 1:** Box-Behnken design and observed experimental data for 3-level–3-factor RSM.

Run	Independent variable ^a^			Extraction yield (mg/g) ^b^		
	*X_1_* (°C)	*X_2_* (%)	*X_3_* (W)	Piceid (*Y_1_*)	Resveratrol (*Y_2_*)	Emodin (*Y_3_*)
1	+1 ^c^ (70)	−1 (40)	0 (120)	9.94 ± 0.57	3.67 ± 0.01	5.75 ± 0.07
2	−1 (30)	−1 (40)	0 (120)	7.99 ± 0.47	3.11 ± 0.26	1.36 ± 0.17
3	+1 (70)	0 (60)	+1 (150)	9.43 ± 0.12	3.73 ± 0.09	10.99 ± 0.13
4	+1 (70)	+1 (80)	0 (120)	7.83 ± 0.12	3.66 ± 0.17	10.96 ± 0.32
5	0 (50)	0 (60)	0 (120)	10.16 ± 0.04	3.72 ± 0.07	9.28 ± 0.02
6	0 (50)	+1 (80)	+1 (150)	7.76 ± 0.02	3.39 ± 0.01	9.57 ± 0.08
7	0 (50)	−1 (40)	−1 (90)	10.32 ± 0.17	3.45 ± 0.01	3.76 ± 0.10
8	−1 (30)	+1 (80)	0 (120)	6.22 ± 0.35	2.95 ± 0.04	7.16 ± 0.30
9	−1 (30)	0 (60)	+1 (150)	8.78 ± 0.07	3.50 ± 0.09	6.56 ± 0.07
10	0 (50)	0 (60)	0 (120)	10.29 ± 0.20	3.70 ± 0.08	9.50 ± 0.04
11	0 (50)	0 (60)	0 (120)	10.37 ± 0.02	3.73 ± 0.14	9.43 ± 0.02
12	+1 (70)	0 (60)	−1 (90)	9.66 ± 0.13	3.70 ± 0.09	10.89 ± 0.22
13	0 (50)	−1 (40)	+1 (150)	10.35 ± 0.12	3.41 ± 0.00	3.63 ± 0.18
14	0 (50)	+1 (80)	−1 (90)	6.07 ± 0.01	2.99 ± 0.16	9.51 ± 0.47
15	−1 (30)	0 (60)	−1 (90)	8.49 ± 0.14	3.10 ± 0.00	5.02 ± 0.10

^a^ Independent variable *X_1_*: Temperature, *X_2_*: Ethanol concentration, *X_3_*: Ultrasonic power; ^b^ Mean of duplicate determinations; ^c^ The values −1, 0, and 1 are coded levels.

The extraction conditions and experimental results of a 3-level, 3-factor Box-Behnken design are shown in Table 1. The manipulated and response variables were analyzed to fit a regression equation (model) that could predict the response within the given range of the independent variables. An analysis of variance (ANOVA) was performed for the model fitted to the experimental data, as given in Table 2. Second-order polynomial equations were given as below for extraction yields for piceid [Equation (1)], resveratrol [Equation (2)] and emodin [Equation (3)], respectively:

Extraction yield of piceid (Y_1_):

Y_1_ = −7.72361 + 0.29948X_1_ + 0.27235X_2_ + 0.05109X_3_ − 0.00227X_1_^2^ − 0.0002145X_1_X_2_ − 0.00343X_2_^2^ − 0.0002704 X_1_X_3_ + 0.00069075 X_2_X_3_ − 0.00030926X_3_^2^ (1)

Extraction yield of resveratrol (Y_2_):

Y_2_ = −1.45753 + 0.04717X_1_ + 0.05436X_2_ + 0.03274X_3_ − 0.00021389X_1_^2^ − 0.00009335X_1_X_2_ − 0.00071135X_2_^2^ − 0.0001522 X_1_X_3_ + 0.0001857 X_2_X_3_ − 0.00013753X_3_^2^ (2)

Extraction yield of emodin (Y_3_):

Y_3_ = −41.6876 + 0.3784X_1_ + 0.8779X_2_ + 0.1293X_3_ − 0.001691X_1_^2^ − 0.0003638X_1_X_2_ − 0.0006017X_2_^2^ − 0.006057X_1_X_3_ + 0.00007782 X_2_X_3_ − 0.0004054X_3_^2^ (3)

**Table 2:** ANOVA for response surface models of all independent variables.

Factor^ a^	Piceid *(Y_1_)*		Resveratrol *(Y_2_)*		Emodin *(Y_3_)*	
	Sum of Squares	Prob > F	Sum of Squares	Prob > F	Sum of Squares	Prob > F
Model	28.56	0.015 *	1.12	0.003 *	130.65	<0.001 *
Linear term						
X_1_	3.61	0.026 *	0.55	<0.001 *	42.70	<0.001*
X_2_	14.38	0.002 *	0.05	0.043 *	64.36	<0.001*
X_3_	0.40	0.345	0.08	0.022 *	0.31	0.267
Quadratic						
X_1_^2^	3.04	0.035 *	0.03	0.111	1.68	0.034*
X_2_^2^	6.95	0.007 *	0.30	0.001 *	21.70	<0.001*
X_3_^2^	0.29	0.419	0.06	0.038 *	0.49	0.178
Interactions						
X_1_X_2_	0.03	0.789	0.01	0.419	0.08	0.544
X_1_X_3_	0.07	0.686	0.03	0.084	0.52	0.167
X_2_X_3_	0.69	0.231	0.05	0.047 *	0.01	0.843
	*R^2^* = 0.94		*R^2^* = 0.97		*R^2^* = 0.99	

^a^ Independent variable *X_1_:* Temperature, *X_2_:* Ethanol concentration, *X_3_:* Ultrasonic power; * Significant at *p*-Value less than 0.05.

The analysis of variance from Table 2 (ANOVA) also indicated that the second-order polynomial model [Equations (1), (2), and (3)] for extraction yields of piceid (Y_1_), resveratrol (Y_2_), and emodin (Y_3_) was statistically significant and adequate to represent the actual relationship between the responses and the variables, with a small model *p*-value (*p* < 0.05) and satisfactory coefficient of determination (*R^2^* = 0.94 − 0.99). From ANOVA evaluation, the overall effects of the three manipulated variables on the response revealed that the temperature (X_1_), and the ethanol concentration (X_2_) were important factors for extraction of piceid, resveratrol and emodin from *P. cuspidatum*. However, ultrasonic power (X_3_) only showed significant effects (*p* < 0.05) on the resveratrol extraction.

### 2.2. Response Surface Analysis

Using surface response plots of the polynomial model, the relationships between the extraction parameters and the response (extraction yield) could be better understood by holding one variable constant and studying the relationship between the other two variables. Since the ultrasonic power showed less effect on *P. cuspidatum* extraction, except for the extraction of resveratrol (Table 2), the ultrasonic power was held constant in the plots. 

**Figure 1:**
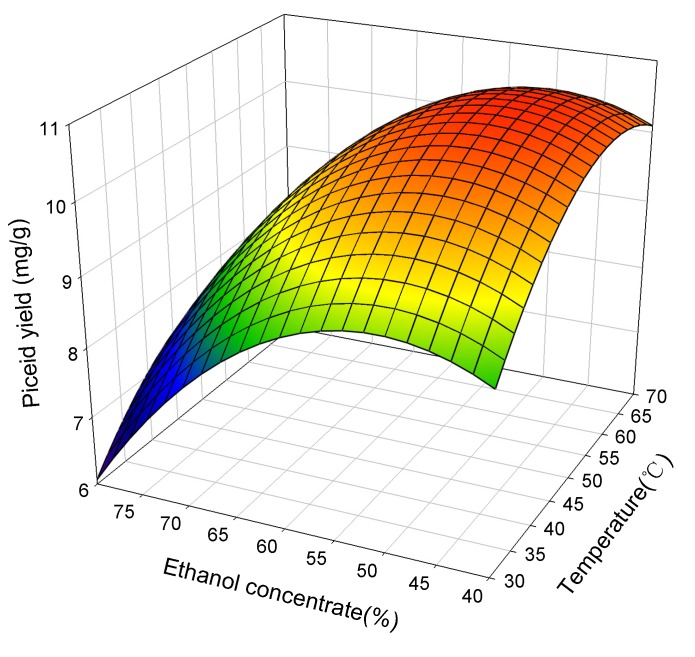
The effect of ethanol concentrate and temperature on the extraction yield of piceid at ultrasonic power of 120 W.

Figure 1 shows the response surface plot for the effect of ethanol concentration and temperature on the extraction yield of piceid at an ultrasonic power of 120 W. A decrease in ethanol concentration from 80% to 50%, with increase extraction temperatures from 30 to 70 °C, enhanced the yield of piceid. With a decrease of ethanol concentration below 50% and an increase of extraction temperature over 60 °C, the yield of piceid gradually leveled off. In other words, the yield of piceid could reach a peak value (~10.25 mg/g) with 40%–50% ethanol concentration and a 55–65 °C extraction temperature. The yield of piceid increased 41.4% as the ethanol concentration decreased from 80% to 40% (at extraction temperatures of 30 °C). In contrast, the yield of piceid only increased 17.6% as the extraction temperature increased from 30 to 70 °C (at ethanol concentrations of 40%), as shown in Figure 1. These results indicate that the effect of ethanol concentration is more significant than temperature on the yield of piceid. It might result from the fact piceid contains a glucoside that increases the polarity, thus the ethanol concentration showed a significant effect on the piceid extraction yield.

**Figure 2:**
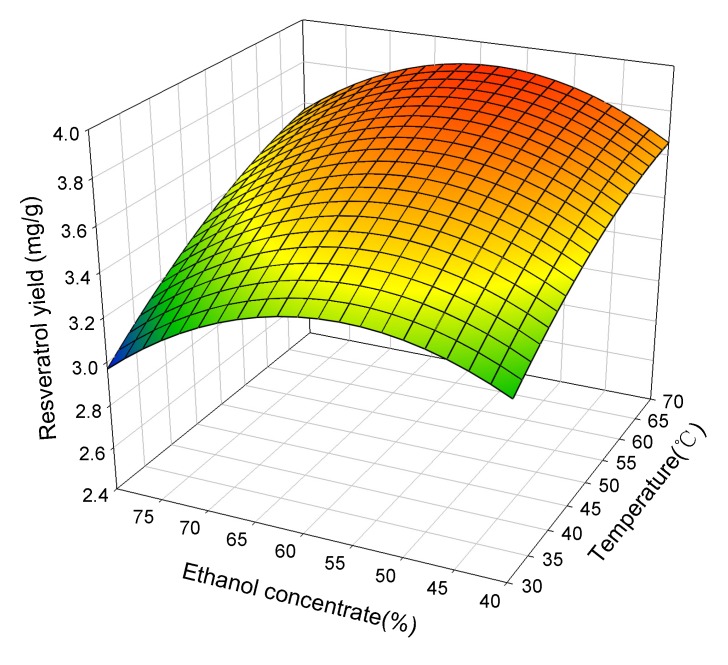
The effect of ethanol concentrate and temperature on the extraction yield of resveratrol at ultrasonic power of 120 W.

Figure 2 shows the response surface plots for the effects of ethanol concentrate and extraction temperatures on the extraction yield of resveratrol at an ultrasonic power of 120 W. A decrease of ethanol concentration from 80% to 60% with an increase in extraction temperatures from 30 to 70 °C enhanced the yield of resveratrol. The yield of resveratrol gradually declined with a decrease in ethanol concentration below 60% at any given extraction temperature. In other words, the yield of resveratrol could reach a peak value (~3.9 mg/g) at 60% ethanol concentration and a 70 °C extraction temperature. The yield of resveratrol increased 14.1% as the extraction temperature increased from 30 to 70 °C (at the ethanol concentration of 40%). In contrast, the yield of resveratrol increased only 7.7% when the ethanol concentration changed from 80% to 40% (at an extraction temperature of 30 °C), as shown in Figure 2. These results indicate that the effect of extraction temperature is more significant than that of ethanol concentration on the resveratrol yield. Since the polarity of resveratrol is less than piceid (trans-resveratrol-3-*O*-glucoside), the effect of the ethanol concentration on the yield of resveratrol was not as significant as that on the yield of piceid. The increasing temperature could improve the extraction yield by enhancing the solubility of the extraction compounds, particularly the non-polar compounds. Also, ultrasonic power had a significant effect (*p* > 0.05) on the extraction yield of resveratrol, as shown in Table 2.

**Figure 3:**
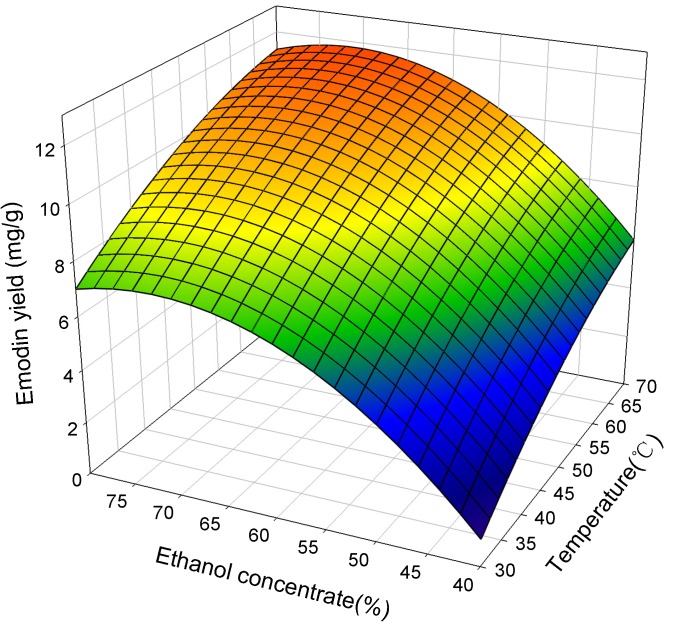
The effect of ethanol concentrate and temperature on the extraction yield of emodin at ultrasonic power of 120 W.

Figure 3 shows the response surface plots for the effect of ethanol concentrate and extraction temperatures on the extraction yield of emodin at an ultrasonic power of 120 W. Increasing the ethanol concentration to 70% and the extraction temperature to 70 °C resulted in maximal emodin yield (over 11 mg/g). On the other hand, decreasing the ethanol concentration to 40% and the extraction temperature to 30 °C resulted in a minimal emodin yield (1.3 mg/g). It was concluded that the ethanol concentration and extraction temperatures were the most important parameters on the extraction yield of emodin and could be considered as indicators of effectiveness.

### 2.3. Attaining Optimum Extraction Condition

The optimum conditions for ultrasonic-assisted extraction of piceid, resveratrol and emodin from the roots of *P. cuspidatum* were obtained from software Design-Expert. As shown in Table 3, the experimental yield under the optimal conditions reached 10.77, 3.82, and 11.72 mg/g for piceid, resveratrol, and emodin, respectively. The predicted results matched well with the experimental results obtained using optimum extraction conditions, which validated the RSM model with a good correlation. Piceid and resveratrol have been extracted from other plant sources, such as grape stems [30], grape berry skins [31], and peanut roots [32], but only in small amounts, ranging from 0.02 to 1.33 mg/g. Our results showed that the extraction of stilbene from *P. cuspidatum* was higher than from other plant sources.

**Table 3:** Optimum conditions for the extraction yield of piceid, resveratrol, and emodin.

Extraction target	Extraction conditions			Predicted Yield (mg/g)	Experimental Yield (mg/g)
	Temp (°C)	EtOH (%)	Power (W)		
Piceid	57.8	49.75	120	10.74	10.77 ± 0.05
Resveratrol	70.0	58.51	120	3.90	3.82 ± 0.06
Emodin	70.0	71.06	120	11.79	11.72 ± 0.11

## 3. Experimental

### 3.1. Materials

Dried *P. cuspidatum* roots were provided by Jing Jiue Co., Ltd. (Taichung, Taiwan) and ground into powder. The powder was screened with a sieve and particle sizes, between 30 and 45 mesh, were used for this study (~0.62 mm). Piceid was purchased from Sigma-Aldrich (St. Louis, MO, USA). Emodin was purchased from MP Biomedicals (Irvine, CA, USA). Resveratrol was purchased from Changsha Nutramax Biotechnology (Changsha, China).

### 3.2. Ultrasound-Assisted Extraction

Powder of *P. cuspidatum* roots (0.1 g) was extracted with 40%–80% ethanol (2 mL) in a cap-sealed glass tube for 20 min. The cap-sealed glass tube was placed in a temperature-controlled 40 kHz ultrasonic bath (Delta DC150H, New Taipei, Taiwan). The internal tank of the ultrasonic bath was 300 × 160 × 150 mm (L × W × H) and ultrasound was input from a flat-plate ultrasonic transducer placed on the bottom of the bath (d = 4.5 cm). The ultrasonic power (90–150 W) was adjusted using a scaled dial disc on the bath panel. Extractions were performed for 20 min under various conditions: temperature ranging from 30 to 70 °C, ultrasonic power from 90 to 150 W, and ethanol concentration from 40% to 80% (v/v). After extraction, insoluble materials were removed by centrifugation at 13,000 rpm for 10 min. The supernatant was analyzed by HPLC.

### 3.3. Experimental Design

The optimal extraction conditions for yielding piceid (Y_1_), resveratrol (Y_2_), and emodin (Y_3_) were determined using RSM. The 3-level, 3-factorial Box-Behnken experimental design was used to investigate and validate extraction parameters affecting the extraction yields of piceid, resveratrol, and emodin. A summary of the extraction temperature, ultrasonic power, and ethanol concentration of the 15 experiments is shown in Table 1. Each experiment was carried out in duplicate. The software Design-Expert (Trial Version 8.0.4, Stat-Ease Inc., Minneapolis, MN, USA) was employed for experimental design, data analysis, and model building. Statistical significance of the model and model variables was determined at a 5% probability level (*p* < 0.05). Three-dimensional response surface plots were generated by keeping one response variable at its optimal level and plotting that against two factors (independent variables). 

### 3.4. Analysis

The three phenolic compounds (piceid, resveratrol, and emodin) were assayed by injecting the extract into an HPLC (Hitachi L-7400; Tokyo, Japan) using a ZORBAX Eclipse XDB C8 column (250 mm × 4.6 mm). The elution solvents used included 0.1% acetic acid in water and methanol. The flow rate was set at 1.0 mL/min. Gradient elution was performed as follows: methanol was set from 10% to 50% for the first 20 min, then the methanol was increased to 100% between 20 and 24 min, and held at 100% for the last 6 min. The UV detector was set at a wavelength of 303 nm. Calibration curves were prepared from piceid, resveratrol, and emodin standards and samples were analyzed by comparing their retention times with those of the standards. 

## 4. Conclusions

The ultrasound-assisted extraction of piceid, resveratrol, and emodin from *P. cuspidatum* roots was successfully developed. The extraction efficiency could be further enhanced by Box-Behnken experimental design and RSM. A second-order model was obtained to describe the relationship between the extraction yields and the parameters of extraction temperature, ethanol concentration, and ultrasonic power. The results indicate that the extraction temperature and ethanol concentration affected the extraction yields of the three compounds significantly, but ultrasonic power only affected the extraction yield for resveratrol. Under optimal extraction conditions, piceid yielded 10.77 mg/g, resveratrol yielded 3.82 mg/g, and emodin yielded 11.72 mg/g. These results show that the ultrasonic extraction technique is a potentially useful method for the extraction of the phenolic compounds from the *P. cuspidatum* roots.
